# Cost of the Diet: a method and software to calculate the lowest cost of meeting recommended intakes of energy and nutrients from local foods

**DOI:** 10.1186/s40795-017-0136-4

**Published:** 2017-03-14

**Authors:** Amy Deptford, Tommy Allieri, Rachel Childs, Claudia Damu, Elaine Ferguson, Jennie Hilton, Paul Parham, Abigail Perry, Alex Rees, James Seddon, Andrew Hall

**Affiliations:** 1 Programme Policy and Quality, Save the Children, 1 St John’s Lane, London, EC1M 4 AR UK; 2 Imperial College, School of Public Health, Norfolk Place, London, W2 1PG UK; 3 London School of Hygiene and Tropical Medicine, Keppel Street, London, WC1E 7HT UK; 4 Department of Public Health and Policy, Faculty of Health and Life Sciences, University of Liverpool, Liverpool, L69 3GL UK; 5 Stainton House, 101 Church Street, Staines, Middlesex, TW18 4XS UK

**Keywords:** Computer software, Linear programming, Food, Cost of the diet, Affordability

## Abstract

**Background:**

When food is available, the main obstacle to access is usually economic: people may not be able to afford a nutritious diet, even if they know what foods to eat. The Cost of the Diet method and software was developed to apply linear programming to better understand the extent to which poverty may affect people’s ability to meet their nutritional specifications. This paper describes the principles of the method; the mathematics underlying the linear programming; the parameters and assumptions on which the calculations are based; and then illustrates the output of the software using examples taken from assessments.

**Results:**

The software contains five databases: the energy and nutrient content of foods; the energy and nutrient specifications of individuals; predefined groups of individuals in typical households; the portion sizes of foods; and currency conversion factors. Data are collected during a market survey to calculate the average cost of foods per 100 g while focus group discussions are used to assess local dietary habits and preferences. These data are presented to a linear programming solver within the software which selects the least expensive combination of local foods for four standard diets that meet specifications for: energy only; energy and macronutrients; energy, macronutrients and micronutrients; and energy, macronutrients and micronutrients but with constraints on the amounts per meal that are consistent with typical dietary habits. Most parameters in the software can be modified by users to examine the potential impact of a wide range of theoretical interventions. The output summarises for each diet the costs, quantity and proportion of energy and nutrient specifications provided by all the foods selected for a given individual or household by day, week, season and year. When the cost is expressed as a percentage of income, the affordability of the diet can be estimated.

**Conclusions:**

The Cost of the Diet method and software could be used to inform programme design and behaviour change communication in the fields of nutrition, food security, livelihoods and social protection as well as to influence policies and advocacy debates on the financial cost of meeting energy and nutrient specifications.

**Electronic supplementary material:**

The online version of this article (doi:10.1186/s40795-017-0136-4) contains supplementary material, which is available to authorized users.

## Background

Undernutrition is a global public health problem that is estimated to be the underlying cause of about 35% of all deaths during early childhood [1]. Although the United Nations has enshrined the right to food in the Universal Declaration of Human Rights, household food security is dependent on two main factors: the availability of food, which may be grown, raised, bought, traded or gathered from the wild; and the physical and economic access to sufficient amounts of food to meet all nutritional needs at all times. While it has been commonplace to blame malnutrition on people’s ignorance of what foods to eat, in circumstances in which foods are available to achieve a nutritious and balanced diet, the main obstacle to access is usually economic [2–5]: people may not be able to afford a diet that meets their needs for energy and nutrients even if they know what foods to eat, or aspire to eat.

With this in mind the Cost of the Diet (CotD) method was developed by Save the Children to apply linear computer programming to select a combination of local foods in amounts that would meet the average needs for energy of one or more individuals as well as their recommended intakes of protein, fat and micronutrients, all at the lowest possible financial cost. The method enables public health nutritionists and food security specialists to estimate the cost and affordability of meeting energy and nutrient specifications using local foods, as the software selects the most nutritious and least expensive. Users can then create models of the effect of interventions such as food subsidies or supplements, or of introducing novel or bio-fortified foods. As a practical tool it could be used to estimate the amount of a cash transfer to meet dietary specifications for example, or to estimate the cost of the additional energy and nutrients needed during pregnancy.

The method was conceived in 2005 and the initial tool, developed in Microsoft Excel © and Microsoft Visual Basic ©, underwent several years of development and testing. In 2013 Save the Children began to redevelop the software in a more stable format and, during that process, reviewed and updated the underlying parameters to provide a coherent scientific basis for the method.

The present paper describes the principles of the method, the mathematics underlying the calculations, the parameters and assumptions on which the calculations are based, and then illustrates the output of version 2 of the software using examples taken from assessments undertaken in a variety of situations. Details of how to obtain the software and a practitioner’s guide in both English and French to use the software, are provided.

## Implementation

The application uses linear programming to calculate the amounts of locally available foods that would need to be consumed to meet specifications for energy, macronutrients and micronutrients for any given individual or group of individuals at lowest possible cost. The application can also include and limit the total weight of each separate food to create a mixture that is similar to local dietary habits. The term ‘diet’ is used in this context to describe the foods selected by the software to meet the recommended intakes of energy and nutrients for a day, week, season or year, but which is limited in all calculations to prevent unrealistic amounts of foods being included and to prevent excessive amounts of some nutrients, to avoid toxicity.

**Fig. 1:**
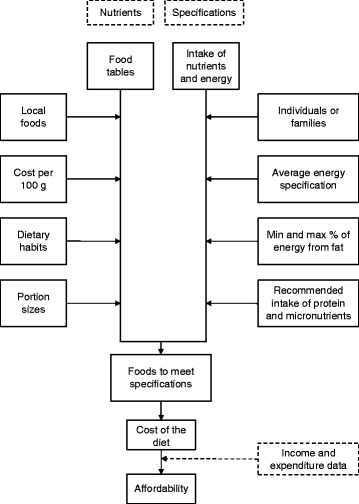
A flow diagram of the information required by the Cost of the Diet software to estimate the cost of meeting specifications for energy and nutrients

Figure 1 shows a flow diagram of the data needed to conduct an assessment, which is separated into two streams: one related to the cost and amount of nutrients provided in foods and the other related to the specification of people’s intakes of energy and nutrients. The information on local foods, their cost per 100 g, and on dietary habits is collected in the location of the assessment using primary data collection methods. The nutrient composition of foods, their portion sizes, and the energy and nutrient specifications of individuals are embedded within the software. If the affordability of the diet is to be estimated, income and expenditure data are required either from a primary source, such as the household economy approach [6], or from a secondary source.

The software applies parameters from five in-built databases: the energy and nutrient content of foods, with the edible portions of raw foods; the energy and nutrient specifications for individuals, sometimes called energy and nutrient ‘requirements’; an optional collection of standard families of between 4 and 10 individuals that are aligned by their average energy intake with families described during a Household Economy Approach [6]; the portion sizes of foods; and currency conversion factors. These parameters are applied to data collected during a survey of all raw foods currently available for sale in local food markets plus some inexpensive processed foods, to obtain the average cost of the edible portion of each food per 100 g. The software then selects foods based on their cost, energy and nutrient content to meet the specifications for energy and nutrients of one or more individuals, but in amounts that can be limited by three factors: the portion size, defined as the maximum weight of any given food that can be eaten in one meal; the maximum weight of all food that can be consumed in one meal; and the number of times each food can be included in the diet in a week, set as minimum and maximum constraints. The basis of the calculations and the data required are described in more detail in the following sections.

### Location and subjects of an assessment

A Cost of the Diet assessment is typically undertaken in an area in which people have a similar diet, such as a province or district, an urban or peri-urban area, an agro-ecological zone or a livelihood zone, especially when a household economy approach (HEA) [6] has recently been done. The links to an HEA are described below, but it is not a requirement of the method. The subjects of a Cost of the Diet assessment are usually the poor, who are reported to buy their foods in small amounts on a daily basis, so may pay a higher unit price than if it was bought in large quantities [7, 8]. The analysis of data for groups with specific dietary habits or needs, such as vegetarians or young children, can be done separately by excluding or selecting specific foods to be included in the assessment.

### Food tables

The software contains a database of the concentration of energy and nutrients in 3,580 food items and supplements, each categorised into one of 15 food groups and drawn from analyses in nine countries. The data are extracted from five main food tables: the WorldFood Dietary Assessment System published by the FAO [9]; a table of foods published by the United States Department of Agriculture (USDA) [10]; a table of foods from West Africa [11]; a table of foods from Bangladesh published by the University of Dhaka kindly included with permission [12]; and a table of average values of common foods calculated for the software, called a CotD food table, described in Additional file 1: Appendix 1. The Bangladesh food tables do not include values for vitamin B_12_ so average values from the other food tables for the same or similar foods were applied. Each entry in the food table also specifies the following: the proportion of each raw food that can be eaten, called the edible portion factor, described in Additional file 1: Appendix 2; a factor to convert liquid foods in millilitres to their weight in grams based on their specific gravity [13]; and iron bioavailability factors [14, 15].

Users may add new foods to the database provided that the edible portion of the food is known and that values for the concentration of energy and all nutrients per 100 g of the food are entered into the main food table that is used by the software to calculate a diet. This function allows users to add new raw foods, processed foods, fortified foods and food supplements, plus data on their cost per 100 g, and so to estimate their impact on the quality and cost of the diet.

### Identifying local foods and seasons

Data on the foods consumed by people in the locality of an assessment are collected in the field. First, a list of locally available raw foods and commonly consumed processed foods is prepared, typically in consultation with local experts or key informants, to include all foods available in all seasons and including all imported, wild and home grown raw foods. Some commonly consumed canned foods such as fish or manufactured foods such as bread may be included, but they may rarely be purchased by the poor, while their cost usually excludes them from the calculations.

Each food is then identified in the food database in the software by selecting either the example that is geographically closest to the assessment site or the generic CotD food, and then transferred to a new screen to create a local food list. The list of foods can then be printed by the software and used to record current and retrospective prices in all seasons during a market survey.

The same local experts are also used to define up to six seasons of the year, depending on local conditions, as the season often affects the availability and price of foods. This is often done as a part of an HEA. The period covered by all seasons must add up to 365 days. This facility could be used to assess the impact of a short term change in the cost of specific foods, for example.

### Market survey of foods: cost per 100 g

A map of the location of all food markets in the assessment area is prepared and an arbitrary minimum sample of six markets are chosen, ideally using a lot quality assurance method of sampling such as centric systematic area sampling method [16], so that markets in the whole area are covered. This serves to take into account the potentially higher cost of foods in small, distant, rural markets compared with markets in large towns.

In each market an arbitrary sample of up to four traders of each food are visited and asked if they are willing to provide information on the cost of foods, ideally during a quiet time of day so that their business is not disrupted. Each trader is asked the price of each item of food, which is recorded, and the weight of three samples are recorded using a battery operated scale with a precision of 1 g such as a Tanita KD 400 (Tanita Corporation, Japan) which weighs items up to 5 kg. This allows for the fact that many fruits and vegetables are sold in quantities that may differ in weight by season but are sold at a fixed price.

If retrospective data on prices are collected, each trader is asked the price of the same food in each preceding season to cover the whole year or, if the price remains fixed, how much of each food is sold in each season, as traders may adjust the amount or number of items sold rather than the price. The collection of retrospective data on prices is less accurate, but can be used to estimate seasonal changes in the cost of a diet during an assessment conducted at a single point in time.

The data on weights and prices are entered from the forms into the Cost of the Diet software which calculates the average cost per 100 g of each food and applies the values to the linear programming calculations.

After the four standard diets have been calculated (see below), users have the ability to alter the cost per 100 g for any or all foods in any or all seasons, by individual or collectively for a group of individuals. This allows an estimate of the impact of a change in food prices due to the season or a shock such as a natural disaster, or estimates of the effect of interventions such as providing free or subsidised food for a given period, for example.

### Currency conversion rates

The price of foods per 100 g can be entered into the software in one of 135 currencies. A database in the software contains average currency conversion rates published by the World Bank for 2013 [17] to allow costs to be calculated in any currency and are convertible into British pounds (GBP), United States dollars (USD) or Euros (EUR). The database will be updated when new values are released. Users can change the conversion factor for any currency or for purchasing power parity [18] and save this in the assessment, although it would override previous calculations.

### Typical dietary habits

An interview is done in the locality of six of the markets with a group of eight to ten local women who prepare food for their family. If an HEA has been done, two women from each wealth group may be selected to assess their potential range in purchasing power.

Each woman is asked how often each week each individual food in the local food list is typically consumed and the answers are categorised to provide a constraint that can then be applied by the software as a maximum and minimum frequency with which each food is consumed in the food habits nutritious diet (see Additional file 1: Appendix 3 and below). For example if most women say that sweet potato is eaten once or twice a day the minimum constraint for sweet potato is set at seven and the maximum is set at 14 so that the software must include sweet potato in the food habits nutritious diet no less than 7 times a week (once a day) but no more than 14 times a week (twice a day). Users can change the minimum and maximum food frequency constraints for individuals or collectively for multiple individuals for each or all foods in any given season. This allows the impact to be assessed of introducing a new food to the diet, to increase or decrease the consumption of any given food, or to exclude specific foods from the diet by setting both constraints to zero.

The same eight to ten women then take part in a focus group discussion to explore whether any foods are taboo for any individuals or in particular periods of the life cycle such as infancy or pregnancy, and to ask if any foods are consumed by specific individuals. This allows individual foods to be excluded from the diet or included for specific individuals.

### Portion size and total weight of food

To ensure that the software allows a realistic amount of food that can be consumed at a meal and to promote dietary diversity, a portion size for each food per meal has been calculated in grams and is applied by the software to each food for each individual in the database by a process of scaling. The portion size specifies the maximum weight of any given food per meal and is adjusted for each individual in proportion to their average energy requirement, a proxy for size.

A standard portion size in grams per meal for all food groups has been estimated for a child aged 1–3 years based upon the recommendation that the maximum percentage of energy should be 50% from carbohydrate foods, 30% from fats, 10% from fruit and vegetables and 10% from protein foods [19]. The exceptions to this are: breast milk, milk powder, supplements, infant foods, sugar, honey, confectionary, herbs, spices, salt, flavourings, condiments, and beverages, as described in Additional file 1: Appendix 9.

The portion sizes of foods for all other individuals are calculated by applying a scaling factor calculated by dividing two standard deviations above the average energy requirement for each individual by the average energy requirement of the child aged 1–3 years. This increases the portion size per meal for the food in proportion to energy for someone with a large average energy specification. The method is described in more detail in Additional file 1: Appendix 9.

Users can adjust the portion sizes for each individual or collectively for all individuals for each or all foods in any given season. This parameter strongly influences dietary diversity and therefore cost, but allows an estimate of the effect of increasing or decreasing the amount in a given portion of food as a result of a programme intervention.

As no data on maximum stomach capacity could be found for all 237 individuals in the database, a uniform, empirical approach has been used to create an upper limit to the amount of food that can be consumed in a day based on the assumption that a given individual with an above average requirement for energy can satisfy their needs from a diet with a relatively low energy density. To calculate this upper limit, the mean plus two standard deviations of the energy specification for each individual has been divided by 1 kcal/g, a diet with a low energy density. The method used is described in more detail in Additional file 1: Appendix 10. This parameter cannot be changed by users at the moment but may in future versions of the software as evidence becomes available.

### Selecting individuals and standard families

The software contains a database of the amounts of energy, protein, fat and 13 micronutrients for 237 individuals specified by the World Health Organization (WHO) and the Food and Agriculture Organization (FAO) [14, 20–22]. Data are specified separately for:

- girls, boys and children of either sex aged between 1–5, 6–8, 9–11 and 12–23 months;
- girls, boys and children of either sex aged between 2 and 18 years in 1-year intervals;
- men aged 18–29, 30–59 or 60+ years with a body weight of between 50 and 90 kg in 5 kg divisions, each for three levels of physical activity, light moderate and vigorous;
- women aged 18–29, 30–59 or 60+ years with a body weight of between 45 and 85 kg in 5 kg divisions, each for three levels of physical activity, light moderate and vigorous;
- and for the additional energy and nutrients specified during three stages each of pregnancy or lactation.

The software allows the selection of an unlimited number of individuals to create a group, such as a family or a household, depending on local definitions. The composition of a family, who typically eat the same foods, can be defined by users based on local knowledge or published data.

Alternatively, if an HEA has been done in the same assessment area, users can select from a database of standard families that are aligned with the HEA in terms of their total average energy requirement, as described in Additional file 1: Appendix 4. Users can choose one of 14 such families consisting of between 4 and 10 individuals. All 14 families include a child aged 12–23 months, a lactating woman and an adult man as standard, plus from one to seven children; seven families also contain a woman aged 60+ years to represent a mother-in-law, plus one to six children. The household members are selected so that the total average energy requirement of the family is N x 2,100 kcal in which N is the total number of individuals in a typical family determined during an HEA, usually by wealth group. These standard families cannot be changed by users but the same method of alignment with an HEA could be applied by users to select another group of individuals for an assessment. This facility allows users to assess the impact of theoretical interventions on vulnerable individuals or groups, or on specific target households.

### Energy specifications

The needs of each individual for energy are specified as the estimated average requirement (EAR) [20] and are applied as a default value to be achieved, but not exceeded, by the software. When this specification is met it indicates that the probability that the energy needs of any given individual are met is 0.5 or 50%.

Users can adjust the default amount of energy for each individual or collectively for all individuals between the 1^st^ and 99^th^ percentile of the EAR. The factors and method used to calculate these values are described in Additional file 1: Appendix 5. This function allows users to calculate the impact on the cost of the diet of increasing or decreasing the probability that energy requirements are met, perhaps if an individual is inactive or very active, or of increasing energy intake during convalescence, for example.

### Protein specifications

The needs of individuals for protein are specified as the 95^th^ percentile of the distribution of requirements per kg body weight [21] multiplied by body weight for each individual, and are applied as a default value to be achieved if possible, but can be exceeded by the software. When this specification is met it indicates that the probability that the protein needs of any given individual are met is 0.95 or 95%. The exceptions are children aged 1–6 months whose recommended daily intake of protein is calculated from the quantity of protein contained in the amount of breast milk required to meet the EAR based on an energy density of 0.67 kcal/ml [23]. This is based on the assumption that the amount of protein in breast milk is sufficient to meet the needs of all infants in this age range.

Users can adjust the default amount of protein for each individual or collectively for all individuals between the 1^st^ and 99^th^ percentile of the recommended intake per kilogram of body weight for all individuals aged >12 months. The factors and method used to calculate these values are described in Additional file 1: Appendix 6. This function allows users to assess how protein specifications influence the cost of the diet.

### Fat specifications

The needs of each individual for fat are specified as a minimum and maximum percentage of their average energy intake, which is converted into grams of fat by applying an energy density of 9 kcal/g, and varies by age group [22]. The software includes in the diet a minimum amount of fat as a percentage of energy depending on age, which is set at 20% for adults, and must not exceed a maximum, which is set at 35% for adults, as recommended by the WHO [22]. The values applied to each age group are given in Additional file 1: Appendix 7. Users can adjust the default amounts of fat for each individual or collectively for all individuals to provide between 1 and 99% of energy from fat for all individuals aged >12 months. This function allows users to assess how fat specifications influence the cost of the diet.

### Vitamins and mineral specifications

The needs of individuals for each of 13 vitamins and minerals are specified as the recommended nutrient intake (RNI) [14] and are applied as default values by the software to be achieved if possible but can be exceeded by the software unless upper limits are set because of toxicity or adverse effects (see below). The default amounts for all micronutrients except vitamin A, which is expressed as a recommended safe intake, are set at two standard deviations above the estimated average requirement. This indicates that the probability that any given individual’s needs are met is 0.97725 or 97.725%.

Users can adjust the default amount of each micronutrient for each individual or collectively for all micronutrients for all individuals to between the 1^st^ and 99^th^ percentile of the RNI. The factors and method used to calculate these values are described in Additional file 1: Appendix 8. The exceptions are pantothenic acid and magnesium, for which no factors are published to allow adjustments, and for all individuals <12 months of age. This function allows users to identify the micronutrients that most influence the cost of the diet and to examine the financial cost of setting the RNI at 2 SD above the mean in order to minimise the risk of deficiency.

### Upper limits for specific nutrients

To prevent the software from creating a diet that exceeds specifications for specific micronutrients that might be toxic, upper limits have been set. The upper limits for vitamin A, vitamin C, niacin, calcium and iron are based upon published evidence of toxicity in excess [14, 24, 25]. The software will not allow these limits to be exceeded so if the limit is met for one nutrient, the specifications for other nutrients may not be reached despite the availability of foods that could provide these missing nutrients. This may mean that the linear programming may not achieve a solution (see below).

### Absorption factors

The absorption from the diet of iron and zinc is reduced by substances such as phytate and oxalate in plant foods, so a proportion of these nutrients are not bioavailable from foods consumed. Published absorption factors have been applied to each food take into account the bioavailability of iron [14, 15] and are described in the table in Additional file 1: Appendix 11. These parameters cannot be changed by users. For zinc, users have the ability to change both the percentile of the RNI and the degree of absorption of zinc from the diet between low, moderate and high bioavailability as defined by the WHO/FAO [14] depending on the quality of the diet of individuals in the assessment area. For example, if households typically consume a diet rich in vegetables containing oxalate or phytate such as spinach and cereals, the bioavailability setting for zinc could be changed from moderate, which is the default, to low, which increases the amount of this nutrient that the software needs to include from foods.

### Standard diets

The Cost of the Diet software applies all parameters for all individuals and foods to a linear programming solver [26] to estimate the lowest possible cost of four theoretical diets. These diets provide standard points of reference for the purpose of comparison and allow the incremental cost of increasingly specific requirements to be estimated. The diets meet the following specifications:

- The recommended average energy requirements of individuals, called an energy-only diet;
- The recommended intakes of energy, protein and fat, called a macronutrients diet;
- The recommended intakes of energy, protein, fat and 13 micronutrients, called a nutritious diet;
- The recommended intakes of energy, protein, fat and 13 micronutrients but limited in amount by the typical dietary habits of households in the assessment area, called a food habits nutritious diet.

### Linear programming calculations

In this context linear programming is a mathematical optimisation process that uses an objective function to minimise the cost of the four diets whilst satisfying constraints for:

- the amount of energy as specified, which is met but not exceeded;
- the proportion of energy from fat, as specified;
- the amounts of protein and 13 micronutrients specified, which is met, but any upper limits are not exceeded;
- the portion sizes, an upper limit to the amount of each food that can be included in the diet;
- the weight of food, an upper limit to the total amount of food consumed;
- the frequency of consuming each food, the number of times each food and each food group can be included in the diet per week.

The programme either establishes a feasible solution, which means that all the constraints listed above are met or adhered to, or an unfeasible solution, which means that a solution that respects all constraints cannot be achieved.

The equations for the cost optimisation and the six constraints listed above are described in detail below. For all following mathematical equations:

i) *X*_*ij*_ represents the weight in grams of food item *‘i’* in food group *‘j’*
ii) The mathematic symbol ∑_*i* = 1_^*r*^ is the sum of all items across all subscript *‘i’* from 1 to *r*.
  For example, ∑_*i* = 1_^3^ A*i* = A_1_ + A_2_ + A_3_
iii) The mathematical symbol ∑_*i* = 1_^*r*^∑_*j* = 1_^*n*^ represents the sum over all subscript *‘j’* from 1 to *n* and all subscript *‘i'* from 1 to *r*

For example:

$$ {\displaystyle {\sum}_{i=1}^2{\displaystyle \kern.6em {\sum}_{j=1}^3\kern.6em {A}_{i j}={\displaystyle {\sum}_{i=1}^2\kern.7em {A}_{i1}+{A}_{i2}+{A}_{i3}=\overset{i=1}{\overbrace{A_{11}+{A}_{12}+{A}_{13}}}+}}}\overset{i=2}{\overbrace{A_{21}+{A}_{22}+{A}_{23}}} $$

Swapping the place of *Σ* does not affect the final answer.

For *X*_*ij*_ defined as above, ∑_*i* = 1_^*r*^∑_*j* = 1_^*n*^ *X*_*ij*_ represents the sum of all weights of food items *‘i’* in food group *‘j’*

The most important function of the linear programming routine is to minimise the total cost of the diet for each individual or group of individuals. The mathematical formula for this function is:

1 $$ \mathrm{Overall}\kern0.5em \mathrm{cost}\kern0.5em ={\displaystyle \sum_{j=1}^r{\displaystyle \sum_{i=1}^{r j}{X}_{i j}\times \kern0.5em  \cos {\mathrm{t}}_{\mathrm{ij}}}} $$

in which: cost_*ij*_ is the cost of food item *‘i’* in food group *‘j’*.

The solver is set to minimise the above expression, which represents the sum of cost for the corresponding amount of each food.

Energy constraints are used to select locally available foods for a diet that provides the estimated average requirements for energy per day, for each specified individual. The software should not create a diet that exceeds or falls below this requirement. The mathematical formula for this function is:

2 $$ {\displaystyle \sum_{j=1}^r{\displaystyle \sum_{i=1}^{r j}{X}_{i j}\times \mathrm{energ}{\mathrm{y}}_{i j}=\mathrm{denergy}}} $$

in which:

i) denergy is the desired total dietary energy content.
ii) energy_*ij*_ is the energy content of food item *‘i’* in food group *‘j’*

Nutritional constraints are used to select locally available foods for a diet that provides the recommended intakes of protein, fat and 13 micronutrients specified by the WHO. These specifications are described as ‘desired’ nutrient specification. The software is allowed to exceed these specifications if necessary but it should not exceed the specific upper limits set for vitamin A, niacin, vitamin C, calcium and iron. The mathematical formulae for the constraints are:

3 $$ {\displaystyle \sum_{j=1}^r}{\displaystyle \sum_{i=1}^{r_j}}\ {X}_{i j} \times \kern0.5em  n u{t}_{i j n\ }\kern0.5em \ge \kern0.5em  d n u{t}_{n\ }\kern1.75em  n\in N $$

4 $$ {\displaystyle \sum_{j=1}^r}{\displaystyle \sum_{i=1}^{r_j}}\ {X}_{i j} \times \kern0.5em  n u{t}_{i j n\ }\kern0.5em \le \kern0.5em  u n u{t}_{n\ }\kern1.75em  n\in N $$

in which:

i) *N* is the set of nutrients of interest.
ii) *n ϵ N* is the nutrient *‘n’* within the set of nutrients *‘N’*.
iii) *dnut*_*n*_ is the desired nutrient requirement for all nutrients *‘n’* of interest in *‘N’*.
iv) *unut*_*n*_ is the upper limit for nutrient requirement for all nutrients *‘n’* of interest in *N*.
v) *nut*_*ijn*_ is the nutrient ‘*n*’ content per gram of food item *‘i’* in food group *‘j’.*

The mathematical formulae for calculating the portion size scaling factor and applying this factor to the standard portion size for an individual are:

$$ \mathrm{Portion}\ \mathrm{size}\ \mathrm{scaling}\ \mathrm{factor}\kern0.5em =\kern0.5em \frac{\mathrm{Mean} + 2\ \mathrm{SD}\ \mathrm{energy}\ \mathrm{specification}\ \mathrm{of}\ \mathrm{individual}}{\mathrm{Mean}\ \mathrm{energy}\ \mathrm{requirement}\ \mathrm{of}\ \mathrm{child}\ 1-3\ \mathrm{years}} $$

$$ \mathrm{Portion}\ \mathrm{size}\ \mathrm{f}\mathrm{o}\mathrm{r}\ \mathrm{individuals}\ \left(\mathrm{g}\right) = \mathrm{Portion}\ \mathrm{size}\ \mathrm{f}\mathrm{o}\mathrm{r}\ 1-3\ \mathrm{y}\ \mathrm{child}\ *\ \mathrm{scaling}\ \mathrm{f}\mathrm{actor}. $$

The number of times per week a portion of food can be included in a diet is limited by applying minimum and maximum food frequency constraints. Using these constraints and the portion size as g/meal for each food, the software calculates the minimum and maximum weekly amount of each food in grams which can be selected for a diet by multiplying the portion size by the weekly frequency. The mathematical formulae for the constraints are:

5 $$ {X}_{ij}\ge m i{n}_{ij}\cdots i=1,2,\dots, {r}_j\kern1em  j=1,2,\dots, r $$

6 $$ {X}_{ij}\le m a{x}_{ij}\cdots i=1,2,\dots, {r}_j\kern1em  j=1,2,\dots, r $$

in which:

i) *min*_*ij*_ is the minimum portion size of food item *‘i’* in food group *‘j’*
ii) *max*_*ij*_ is the maximum portion size of food item *‘i’* in food group *‘j’*

The number of times per week a food from any given food group can be included in the diet is limited by applying a maximum food group constraint. This enables the user to adjust the frequency with which each food group can be consumed in a week. For all diets the maximum frequency is set at a default value of 105 times per week for all food groups. This gives the software the option to include up to five foods from a food group for three meals a day, 7 days a week. The mathematical formula for this function is:

7 $$ {\displaystyle \sum_{i=1}^r}{\displaystyle \sum_{j\in S(s)}^{r_j}}\ \frac{X_{i j\ }}{a{ v}_{i j\ }}\ \le \kern0.5em  fgma{x}_s $$

in which:

i) *av*_*ij*_ is the weight (in grams) of an average portion size of the specified food item ‘*i*’ in food group ‘*j*’.
ii) *S(s)* is the food group ‘*s*’ within the set of food groups *S*.
iii) *fgmax*_*s*_ is the maximum number of servings in the food group ‘s’.

The total quantity of food (in grams) that the software can include in a diet is limited by applying a total food weight constraint. The mathematical formula for this constraint is:

8 $$ {\displaystyle \sum_{j=1}^r}{\displaystyle \sum_{i=1}^{r_j}}\ {X}_{i j\ }\ \le\ \mathrm{T}\mathrm{F}\mathrm{W} $$

in which TFW is the total food weight.

### Cost of the Diet software

The design specified by Save the Children for the Cost of the Diet application determined the achitecture of the software. The specifications were: the application should be deployable without requiring any other software to function and should not require administrator rights to install it; the software should need only to be copied onto a hard disc from which it should run directly; and the software should run on low specification computers using a Microsoft Windows operating system from Windows XP to the latest version available in 2016.

The software is a database application using the Windows Single Document Interface (SDI) model. The user interface has been implemented to use a “wizard” type workflow whereby the user navigates backwards and forwards through a series of screens, building an assessment model on the way. Each screen is presented in the form of a spreadsheet with both data and most of the navigation links. A menu structure has been implemented to make the navigation familiar to Windows users, so it is intuitive.

The application has been written in Embarcardero’s Delphi (version XE7 ©) an object-orientated language based on Object Pascal. This was chosen because of Delphi’s reputation for rapid application development, its ability to be built with little modification for several platforms, and that fact that it runs independently without the need for installation. The application has three major components:

1. The user interface to enter data, to navigate through the system and to select reports, which includes third party software TMS Grid Pack © matrix utilities and FastReport © report generator.
2. The linear programming module (lp_solve version 5.5.2.0) that optimises the foods chosen within diets to minimise cost and within chosen dietary constraints [27].
3. The database back-end (SQLite) that stores all the data entered by the user to build the assessment, manipulate the data to feed into lp_solve, and store results for presentation.

The software can be downloaded from: http://www.heacod.net/countries/reports/cotd-software-version-2-2016/ Users are asked to register and agree to the licence conditions to use the software, which is free. Registration ensures that users will be informed of updates to databases and bug fixes. Bugs should be reported to cotd@savethechildren.org.uk with a screen capture that includes any error message. The method and software are explained in a manual [28], which is available in English (http://www.heacod.net/countries/reports/cotd-practitioners-guide-v2-english/) or French (http://www.heacod.net/countries/reports/cotd-practitioners-guide-v2-french/).

### Income and expenditure data

The cost of a nutritious diet becomes a more meaningful figure when compared with the income and essential expenditure of the poorest members of the community in which an assessment is done. A diet may be inexpensive in comparison with other contexts, but if it is beyond the means of the poor, then a risk of malnutrition exists. If an analysis is wanted of the affordability of the diets generated by the Cost of the Diet software, then information can be entered into the software on the annual income and non-food expenditure for one or more wealth groups. This allows an estimate to be made of the impact of an income generating activity or a social protection scheme such as a cash transfer, on the affordability of a nutritious diet. Such data are usually available from an HEA, a livelihoods based analytical framework which is designed to provide an estimate of household economy for arbitrary levels of wealth within a given community [6]. An HEA also provides useful contextual data such as: the division of a region into livelihood zones; the location of markets and villages; the division of the population into wealth groups; the typical annual income and essential non-food expenditure by wealth group; the typical household size by wealth group; the sources of food for households including wild foods; and a seasonal calendar [6]. For these reasons the two tools have been aligned as described in Additional file 1: Appendix 4. If an HEA has identified that different wealth groups typically contain a different number of individuals within a household, then the cost of the four standard diets will need to be calculated for each of the household sizes to ensure the estimates of affordability are accurate.

## Results and discussion

The aim of the Cost of the Diet software is to identify a mixture of foods that meets the recommended energy and nutrient specifications for any given individual or group of individuals within the limits and constraints outlined in the previous section, at the lowest possible cost.

### Output of the Cost of the Diet software

The results are presented in the form of tables and graphs that show for each of the four standard diets the cost and degree to which the specifications can be met using locally available foods. Every graph produced by the software can be exported into Microsoft Word© while all tables of data can be exported into either Word© or Excel© for editing if necessary.

Table 1 summarises the outputs of the software for each of the four standard diets for any given individual or selected group of individuals by day, week, season and year. These four standard diets apply the default values of all parameters in order to provide a basis to compare the results from different assessments and must be calculated first, before any changes are made to the parameters. The four diets are incremental: by adding specifications and placing restrictions on the frequency and amounts of each food, a mixture of foods is created that is more typical of a diet, so the cost typically rises. For example, the energy-only diet is the least expensive because the software needs only to meet the specification for the average energy intake of the individuals selected. The food habits nutritious diet is usually the most expensive because all the nutrient specifications need to be met without exceeding any upper limit, while constraints are imposed on the frequency and amounts of foods that can be added in order to create a mixture of foods that is similar to the typical food habits of the population in the assessment area. These limits act to increase the number of foods in the diet and, once the maximum weight of each food has been reached, foods of higher cost or lower nutritional quality are selected to meet the specifications.

**Table 1:** A summary of the data presented in tables by the Cost of the Diet software for each of the four standard diets. Y = Yes, N = No

Output of software	Day	Week	Season	Year	Individual	Family or HH*
Cost of each diet in currency units	Y	Y	Y	Y	Y	Y
Cost of each food included in each diet in currency units	Y	Y	Y	Y	Y	Y
Cost of a food as percentage of the total cost of each diet	N	N	Y	Y	Y	Y
Weekly cost of food groups in currency, for food habits nutritious diet only	N	Y	Y	Y	Y	Y
Number of foods included in each diet	Y	Y	Y	Y	Y	Y
Number of servings of each food in each diet	Y	Y	Y	N	Y	Y
Number of food groups included in each diet	Y	Y	Y	Y	Y	Y
Edible weight of each food in each diet in g	Y	Y	Y	Y	Y	Y
Total weight of each food in each diet in g	Y	Y	Y	N	Y	Y
Quantity of a food included in a diet as a percentage of total edible food weight	N	N	Y	Y	Y	Y
Quantity of nutrients provided by the edible portion of food in a diet in g	Y	Y	Y	N	Y	Y
Nutrients provided by each food as a percentage of the target specifications	Y	Y	Y	Y	Y	Y
Percentage of energy and nutrient specifications met by each diet	Y	Y	Y	Y	Y	Y
Affordability of diet as percentage of income by wealth group, if income and expenditure data entered (not for macronutrients diet)	N	N	Y	Y	Y	Y

*HH = household

The food habits nutritious diet may be less expensive than the nutritious diet if the software is not able to calculate a nutritious diet when typical dietary habits are imposed because a mathematical solution is not achieved. This indicates that local food habits, perhaps influenced by economic poverty, food taboos or food preferences, may affect the inclusion of nutritious foods in the diet. For example, if poor people say that they do not eat eggs, for whatever reason, the constraints should be set so as not to allow the software to include eggs in the diet, even if they are available.

Any comparisons of costs within or between assessment areas are valid only when all the specifications for a diet have been met. If any specification is not met, the total cost is therefore incomplete and costs cannot be compared.

Table 2 illustrates how data are displayed by the software on the weight of 1 day’s food for a nutritious diet in a given season for each of seven members of a household. The table shows the edible weight of each food for each individual and in total, and the total weight of raw food that the household would need to purchase in the market. In this instance the software selected 18 foods from seven food groups, including Bengal gram, milk and three different varieties of green leafy vegetables.

**Table 2:**
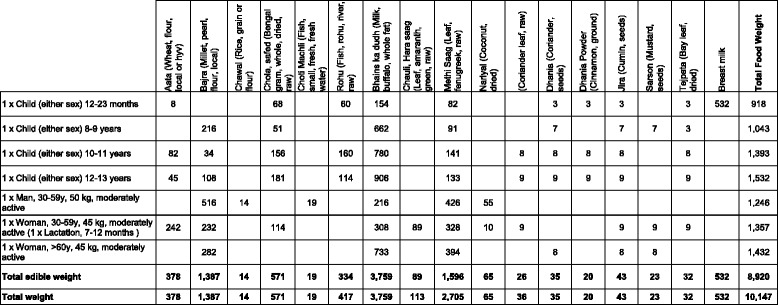
An example of a table produced by the software showing the foods by weight in grams selected for 1 day for a household of seven individuals for a nutritious diet from an assessment in Pindra block, India

Table 3 illustrates how data are displayed by the software on the percentage of energy and nutrients for each food for a day in a given season for a food habits nutritious diet. The table shows that dried fish has been selected as an inexpensive and rich source of protein, vitamin B_2_, niacin, pantothenic acid, vitamin B_12_, calcium, iron, magnesium and zinc. Lentils have been selected by the software as an inexpensive source of vitamin B_1_, vitamin B_6_ and folic acid, and provide most of these nutrients in the diet. The software can produce a table of the same structure to summarise data for a week, again for each season.

**Table 3:**
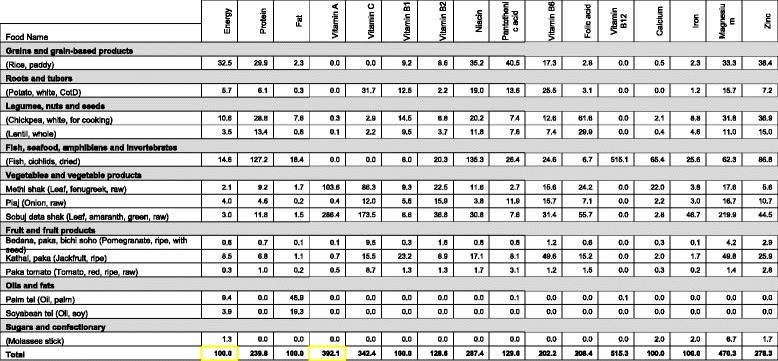
An example of a table produced by the software showing the foods selected for 1 day and the percentage of energy and each nutrient that is provided by each food for a household of eight individuals for a food habits nutritious diet from an assessment in in Sylhet, Bangladesh

Table 4 illustrates how data are consolidated and displayed by the software for a period of a year for a hypothetical family of seven individuals. The table shows the total edible weight and cost of the foods selected by the software for a food habits nutritious diet for a year with the percentage contributed by each food in terms of weight, cost, energy, protein and fat; the percentage contribution of each food for eight vitamins and four minerals; and the percentage of the total target met for each nutrient, for all seasons combined. In this example the software has selected eggplant leaves as an inexpensive source of B-group vitamins, vitamin C, vitamin A and calcium, while small dried fish and liver have been selected as rich sources of vitamin B_12_.

**Table 4:**
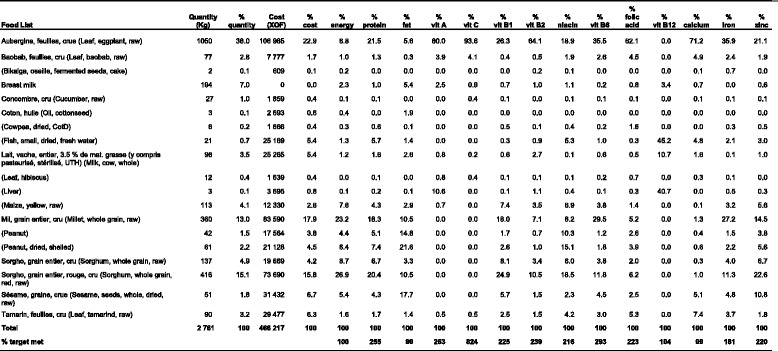
An example of a table produced by the software that shows the foods selected for a period of year for a food habits nutritious diet for a household of seven individuals from an assessment in Kaya, Burkina Faso, the edible weight and cost of each food, and the percentage of energy and each nutrient that is provided by each food

**Fig. 2:**
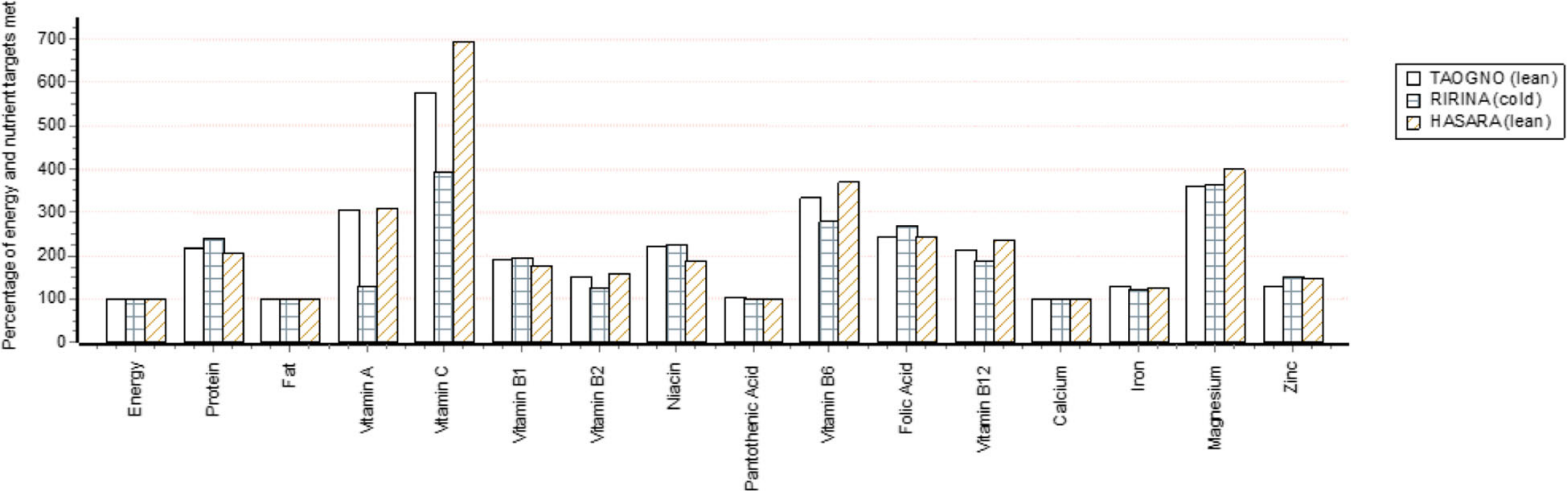
An example of a graph produced by the Cost of the Diet software for a food habits nutritious diet from an assessment for a hypothetical family of six individuals in Sava, Madagascar showing the percentage of the specifications met for energy, macronutrients and micronutrients in each of three named seasons in a year

The data presented in Tables can also be displayed by the software in the form of simple graphs. Figure 2 is an example for a hypothetical family of six individuals for a food habits nutritious diet and shows that the specifications for all nutrients for which the software has met by 100% or more in all three seasons. The specifications for some micronutrients may exceed 100% as some have no upper limits. This occurs when amounts of foods are included by the software to meet the specification for another, less available micronutrient. For example, vitamin C, folate and B-group vitamins are often found in green leafy vegetables that contain carotenoid pigments that are converted into vitamin A. When the amount of a nutrient is met by exactly 100% it indicates that this specification is the hardest for the software to meet from locally available foods, but that a solution has been achieved. Any percentage <100% indicates that a solution has not been reached. This does not necessarily mean that the diet is not nutritionally adequate for some individuals, just that the high specification set by the WHO/FAO for the recommended nutrient intake [14] has not been met.

There are at least four reasons why the software cannot meet specifications for one or more nutrients.

The first is that foods are not available that can provide the nutrient in sufficient quantities, perhaps because the foods are seasonal. This outcome can occur for the nutritious diet, which is allowed to include any combination of foods for up to three meals a day in amounts limited only by their total weight, not by portion size, to meet nutrient specifications.

The second reason is that typical dietary habits, perhaps influenced by economic poverty, food taboos or food preferences, restrict the number of foods containing specific nutrients that can be included in the food habits nutritious diet. If the target for a particular nutrient is met by 100% or more in the nutritious diet but is met by less than 100% in the food habits nutritious diet, then this indicates that typical dietary habits are restricting the amount of a food or foods that the software can include to provide this nutrient. This could be because of food preferences or taboos, or because households cannot afford to buy the foods, both of which mean that specific foods should be excluded from the possible diet by setting the number of times the food can be eaten to zero. This can also be done for foods not given to specific individuals, such as very young children.

The third reason could be a combination of both availability and typical dietary habits, in which case the specification for a nutrient will not be met in either the nutritious diet or food habits nutritious diet.

The fourth reason is that an upper limit has been reached for a specific nutrient before the specifications for all individual nutrients have been met, so the software cannot add any more foods that would contribute to exceeding that limit. Upper limits are set for energy, for some micronutrients, and for the maximum weight of food that be consumed in a meal, as described above. If the upper limit for energy or a nutrient has been met, the software will flag the total percentage of energy or the specific nutrient with a yellow border in the daily or weekly report (see example in Table 3). If the upper limit for the weight of food has been reached, the software will display a warning: *‘This diet cannot be calculated, because an upper limit was reached, this will be either food weight or one of the nutrients*’.

**Fig. 3:**
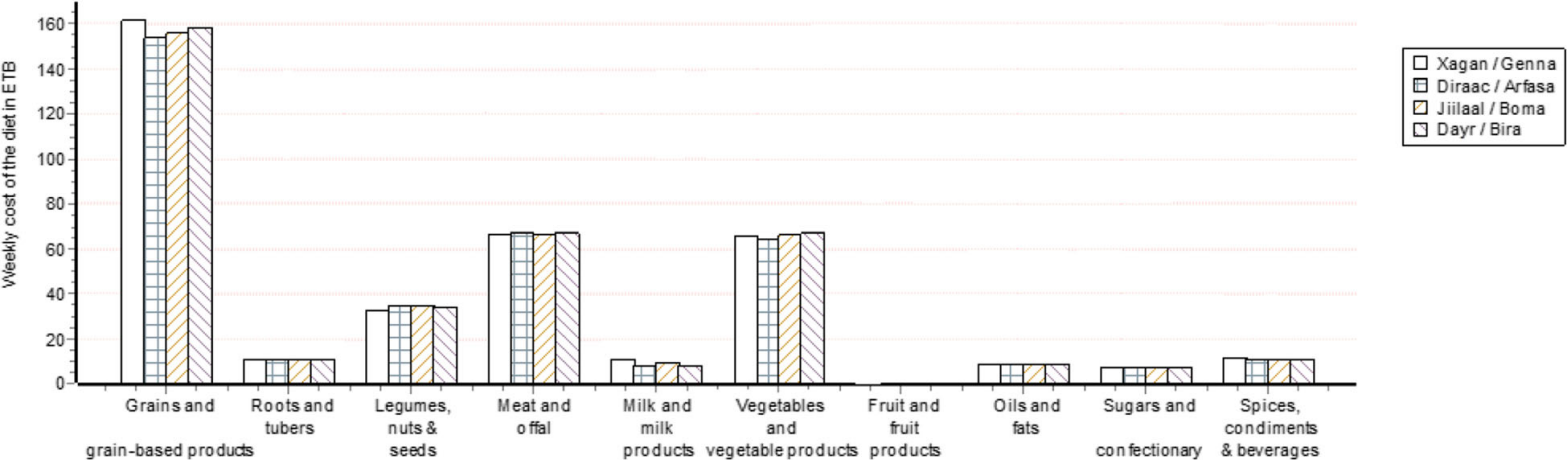
An example of a graph produced by the Cost of the Diet software for a food habits nutritious diet from an assessment for a hypothetical family of five individuals in Dessie, Ethiopia showing the cost per week in Ethiopian Birr of the food groups in each of four named seasons in a year

Identifying the food groups that contribute the most to the cost of a food habits nutritious diet is another useful way of emphasising the cost, nutrient targets and composition results for this diet. Figure 3 illustrates how the software summarises these data using an example for a household of five individuals in which milk and fish products contribute most to the cost of a food habits nutritious diet because they are the main and least expensive sources of calcium, one of the most difficult nutrient specifications to meet.

The affordability of the energy-only, nutritious and food habits nutritious diets, plus essential non-food expenditure, can be shown as graphs by season and year for individuals or households. This requires data on annual income and essential non-food expenditure for any wealth group, although normally a Cost of the Diet assessment will estimate affordability of each diet for the four wealth groups identified during an HEA, typically described as very poor, poor, middle and better-off wealth groups. These are relative categories, not absolute.

**Fig. 4:**
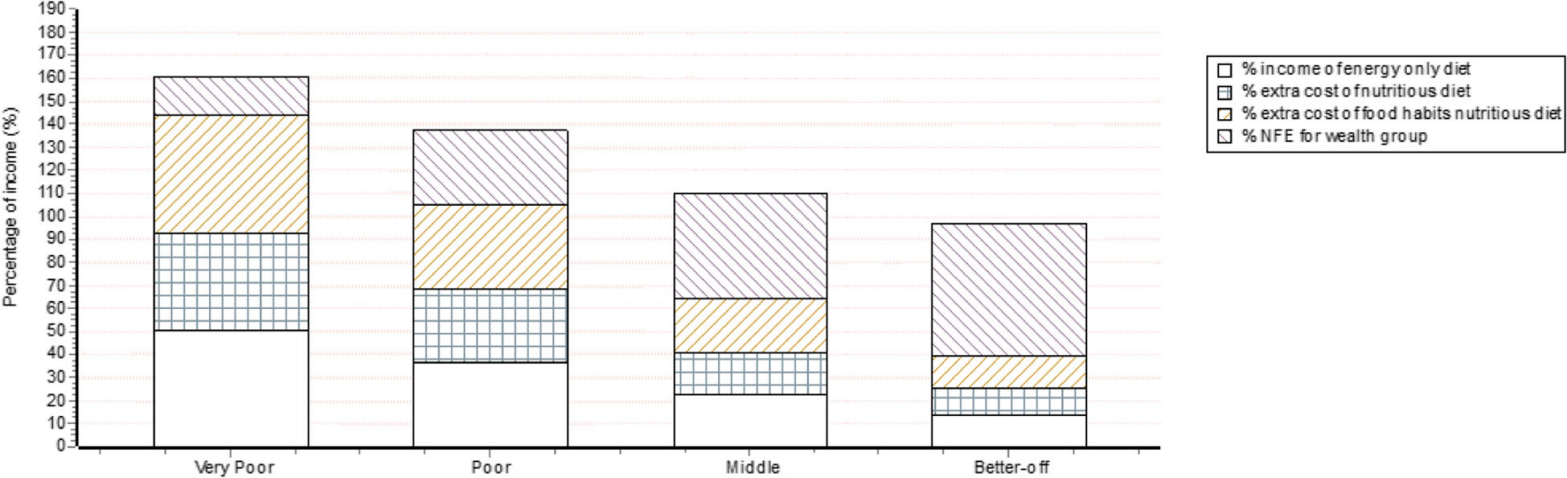
An example of an affordability graph shown as percentage of income produced by the Cost of the Diet software from an assessment for four wealth groups in Shikarpur, Pakistan. Income and non-food expenditure (NFE) data have been estimated during a Household Economy Approach

Figure 4 presents an example for four wealth groups consisting of a household of eight individuals, and shows the cumulative percentage of income that would be spent on the energy-only, nutritious and food habits nutritious diets, plus non-food expenditure. This can be used to estimate the percentage of households that might not meet their energy and nutrient specifications after essential non-food expenditure is met, and to estimate the amount of money required to close the gap between income and the cost of each of the three diets. Affordability is not calculated for the macronutrients diet.

### The effect of changing underlying parameters

Version 2 of the Cost of the Diet allows users to change almost all of the parameters applied by the software to:

- Add foods and supplements;
- Change the price of any food or make it free;
- Change the portion sizes of foods;
- Change the minimum and maximum number of times a specific food or a food group is consumed in a week;
- Select any number of predefined individuals to create groups such as families or households;
- Change the amount of energy provided by fat to between 1% and 99%;
- Change the amounts of energy, protein and micronutrients from the 1^st^ to the 99^th^ percentile of specifications for any given individual or collectively for a group of individuals;
- Add or delete wealth groups and change their annual income and annual non-food expenditure.

Once the standard diets have been calculated using the default parameters, models can be created so that, by adding foods or changing parameters, the potential effect on the cost of the diet, on dietary diversity and on nutrient sufficiency can be modelled for a wide range of theoretical interventions for individuals, families or households.

For example: add novel, rare, unusual, improved or fortified foods to the diet [29]; examine the impact of supplements on the cost of meeting nutrient specifications; support households with food security or livelihoods interventions to increase dietary diversity, food intake or income; test the potential impact of shocks that affect the prices of food or decrease food availability; identify the most nutritious food in a food group by equalising the cost per 100 g to see which foods are included in the diet; test the effect of improved child feeding practices on the cost, composition, quality and affordability of a nutritious diet for children older than 6 months. This list is not exhaustive and users may identify novel uses for the software.

The results that the Cost of the Diet produces are based on the values of the underlying parameters that are applied by the linear programming solver. Changes to these parameters can greatly influence the results that the software produces. In particular, the portion sizes play a key role in determining the number of foods and the cost of the diet. When the portion size is relatively large (>100 g) the software can meet nutrient specifications from a few foods, the dietary variety is small and the cost is minimised by including relatively large amounts of the least expensive foods. When the portion size is small (20 g) then the software is forced to add additional foods in similarly small amounts, some of which are more costly than the least expensive but most nutritious alternatives. This parameter thus increases the diversity of the diet and increases the cost, as more expensive foods are included in the solution.

**Fig. 5:**
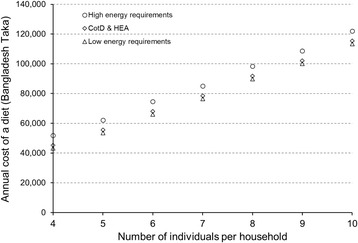
The range in the annual cost of a nutritious diet for families of between four and 10 members depending on how their energy specifications are set. The Cost of the Diet and Household Economy Approach (CotD & HEA) family is adjusted so that the average energy specification is as close to 2,100 kcal/person as possible, the basis of all calculations in an HEA

The number and choice of individuals to include in a family also has an impact on the cost of the diet produced by the software. Figure 5 illustrates the potential range in cost in Bangladesh Taka (BDT) using an example from an assessment. It shows the annual cost of a nutritious diet for the seven standard CotD and HEA families of between four and ten members with an average energy specification of 2,100 kcal per person, the basis of the calculations in an HEA [6]. The costs are also shown for two other families with the same number of members but specified to cover the highest and lowest possible energy specification for all individuals in each hypothetical family. The minimum or low energy family was selected by choosing the lightest and least active family of between four and ten individuals, while the maximum or high energy family was selected by choosing the heaviest and most physically active family of between four and ten individuals. Figure 5 shows that by selecting different individuals to create a hypothetical family with the same number of individuals the cost of a nutritious diet could be 6,570 BDT (14.5 to 5.7%) higher than the cost of a CotD/HEA diet for all high energy requiring families, to 1,970 BDT (4.4 to 1.7%) lower for all low energy requiring families. The fact that households contain more individuals could include an effect of economies of scale when purchasing food, especially staples. This effect could also be modelled in the software by reducing the price per 100 g of foods, but the unit cost of buying in bulk to achieve a lower unit price should be recorded during the market survey.

**Fig. 6:**
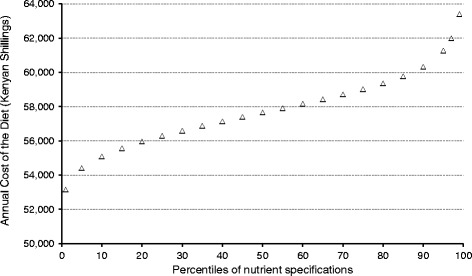
The annual cost of a nutritious diet from an assessment for a hypothetical family of seven individuals in Turkana, Kenya when the WHO/FAO specification for calcium is varied between the 1^st^ and 99^th^ percentile of the recommended nutrient intake mostly in increments of 5 percentile points

The ability to set the amounts of energy, protein and micronutrients to any value between the 1^st^ to the 99^th^ percentile of specifications allows the effect of the WHO/FAO recommended nutrient intake to be assessed. This is set for most micronutrients at 2 SD above the mean, equivalent to the 97.725^th^ percentile, to minimise the rise of deficiency in a population, but is an amount which probably exceeds the actual needs of >95% of all individuals. Figure 6 shows the impact that changing the specifications for calcium has on the cost of a nutritious diet for a hypothetical family of seven individuals. The curve is sigmoidal in shape and shows that above the 85^th^ percentile, the annual cost of the nutritious diet increases exponentially as the software includes more, expensive foods to meet the higher specification for calcium. There was no effect on cost when adjusting the amounts of any other micronutrients, so the cost of the diet was driven by the specifications for calcium. This facility could be used to determine the amount of a cash transfer to buy foods that meet micronutrient specifications with a less stringent probability, or to estimate the cost of a high energy specification perhaps due to the needs of greater physical activity or in convalescence.

### Comparison with other software

The use of linear programming optimisation to create mixtures of foods is not a new method in animal [30–32] or human nutrition [33–35]. Optifood software is similar to the Cost of the Diet in that it applies linear programming to formulate and test population-specific food-based recommendations to meet the nutritional needs of specific human individuals [36]. Whilst the Cost of the Diet software produces a hypothetical diet for individuals or families based upon the lowest cost combination of all available foods, Optifood aims to formulate food-based dietary guidelines using foods currently eaten or acceptable to the target population based on observed portion sizes and 24 h or longer recalls of foods eaten [37, 38].

### Limitations of the method and software

The starting point of the Cost of the Diet software is not the diet of individuals, so it is not a tool to plan a diet nor does it analyse the nutrient content of the foods in a given diet. The software determines the amounts of the least expensive, locally available foods that meet the energy and nutrient specifications of selected individuals or a group of individuals such as a family. Depending on the foods available and their cost, the software may not include animal source foods if nutrients can be obtained less expensively from plant foods. In these circumstances, animal source foods would need to be included purposefully by setting the number of times a food is to be eaten in a week.

The software does not take into account the distribution of foods within a household, which may be unequal. The software does not take into account the loss of nutrients that occur during cooking, though these may be covered to a degree by the high specifications set for micronutrients. The software does not include in its calculations the needs of individuals for iodine and vitamin D because no values for their concentrations in food are published in food tables. The software also does not calculate needs for essential fatty acids or amino acids because such data for foods are not commonly available and because no daily intakes are recommended by the WHO.

The software does not distinguish between native substances with retinol activity and retinol derived from beta-carotene and other carotenoids. Users may therefore find that in diets in which the software has selected green leafy vegetables and orange flesh fruit and vegetables to provide vitamin A, the upper limit for vitamin A may be reached within the software, even though these foods contain beta-carotene, which is not toxic in excess, unlike native retinol from animal products such as liver. This issue will be rectified in a future version of the programme.

### Future development of the Cost of the Diet tool and software

Version 2 of the software was rewritten in Delphi ® to provide a platform on which to develop it further and include new functions, dependent on funding. These may include versions in a language other than English and the ability to import food price data from handheld devices to which the market survey forms can be exported. Another facility that may be added is the ability to express the amounts of micronutrients provided by the mixture of food as percentiles of the RNI as well as percentages of the RNI, as they are substantially different and not linearly related. Users are asked to propose additional functions and improvements to cotd@savethechildren.org.uk.

## Conclusions

The Cost of the Diet is a tool to develop thinking and stimulate debate about foods, nutrient sufficiency and nutrition security. The flexibility of the software to change the underlying parameters gives the potential to understand what nutrients drive the cost of meeting the RNI in any given locality and to examine the potential effects of changes in food availability and the importance of economic access to nutritious foods. The underlying food and nutrient databases are useful reference material.

The results from a Cost of the Diet assessment could be used in conjunction with other contextual information and data from nutrition and food security surveys to inform nutrition, food security, livelihoods and social protection programmes delivered by development agencies; to inform and influence nutrition and food security related policy; and to inform advocacy processes and debates. Conducting periodic market surveys to collect data on the prices of the relatively small number of foods selected by the software in an assessment as they change by season or due to shortages could potentially enable the tool to be used as an indicator within food security and nutrition early warning systems, although this has not been tested. Data on food prices could be provided each season or periodically by traders who send a text message on a mobile telephone for a small reward of credit, so an assessment could be updated regularly and at low cost, and changes in the cost of the foods selected by the software could be tracked over time.

The description of the method in the present paper and the release of the free software developed by Save the Children will allow practitioners to undertake standard Cost of the Diet assessments and describe novel applications of the method. This could contribute to a new body of knowledge on the actual financial cost in both developing and developed countries of meeting human energy and nutrient specifications from inexpensive, locally available foods.

## Additional file

Additional file 1: A text file containing 11 appendices describing specific aspects of the software referred to in the main text of the paper. (DOC 94 kb)

## Abbreviations

CotD: Cost of the Diet
EAR: Estimated average requirement
EUR: European Union euros
FAO: Food and Agriculture Organization
GBP: Great Britain pounds
HEA: Household Economy Approach
RNI: Recommended nutrient intake
USD: United States dollars
USDA: United States Department of Agriculture
WHO: World Health Organization
