# Supplementary material for: Cost of the Diet: a method and software to calculate the lowest cost of meeting recommended intakes of energy and nutrients from local foods
Source: BMC Nutr. 2017 Mar 14;3:26. doi: 10.1186/s40795-017-0136-4 (PMC7050783; doi:10.1186/s40795-017-0136-4)
Supplement: Supplementary file 1 — A text file containing 11 appendices describing specific aspects of the software referred to in the main text of the paper. (DOC 94 kb) [file 40795_2017_136_MOESM1_ESM.doc]

**Appendix 1.** *Generic foods database*.

Because the WorldFood Dietary Assessment System database contains several examples of the same foods, such as rice, wheat and maize, each with slightly different nutrient values, a generic CotD food composition table was created by calculating average values from all examples of 28 of the most common foods listed in the database. These CotD foods can be used when no country-specific data are available or for comparing analyses between countries. Each food is identified in the food table by the abbreviation CotD after the name of the food.

**Appendix 2.** *Edible portion sizes.*

These factors tell the software the proportion of each raw food that is edible. For example if an estimated 69% of a whole banana is edible, the edible portion size factor is 0.69 so:

Edible portion = raw weight of food (g) * edible portion scaling factor

This factor is applied to each raw food when calculating how much energy and nutrients it provides in the raw form and have been taken from the Bangladesh and West Africa food composition tables. The tables of results produced by the software show the weight of edible food that is calculated to meet energy and nutrient specifications and the weight of each raw food that needs to be bought in the market.

**Appendix 3.** *Diets analysed by the Cost of the Diet software.*

The software calculates for an individual or group of individuals the cost of four standard diets, each of which is incrementally better in term of quality and realism. These diets provide standard points of reference to allow comparisons to be made within and between assessments, before any parameters are changed by users.

Energy-only diet. This diet aims to meet the average energy specification, at lowest possible cost. The analysis is not used to promote an energy-only diet because it could lead to micronutrient deficiencies, but allows the incremental cost of meeting micronutrient specifications to be estimated.

Macronutrients diet. This diet aims to meet the average energy specification and the recommended intakes of protein and fat as a percentage of energy, at the lowest possible cost. This diet could also lead to micronutrient deficiencies but allows the incremental cost of meeting recommended micronutrient specifications, in addition to energy, protein and fat, when other diets are calculated.

Nutritious diet. This diet aims to meet the average energy specification and the recommended intakes of protein, fat as a percentage of energy, and 13 micronutrients, at lowest possible cost. This diet does not reflect people’s typical dietary patterns as large amounts of a small number of foods may be included each and every day in the assessment period, but it illustrates the incremental cost of meeting specifications for micronutrients when compared with the energy-only or macronutrients diets. When compared with the food habits diet below, the nutritious diet is useful to illustrate the differences in the number of foods selected and their cost, when the software takes into account typical dietary patterns.

#### Food habits nutritious diet. This diet aims to meet the average energy specification and the recommended intakes of protein, fat as a percentage of energy, and 13 micronutrients whilst applying lower and upper limits to the number of times a week that each food can be included in the diet to reflect typical dietary habits, all at lowest possible cost. The lower limit ensures that a food is included in the diet a minimum number of times a week while the upper limit is set by the maximum, based on three meals a day, 7 days a week. This diet illustrates the incremental cost of ensuring that the diet takes into account typical dietary habits such as the main staple consumed, the frequency with which foods are eaten, and food taboos.

**Appendix 4.** *Individuals and standard families*.

The Cost of the Diet software can select a family that is aligned by their total energy specification with the number of individuals identified during the Household Economy Approach (HEA), a livelihoods based framework designed to provide an estimate of the household economy of different wealth groups in a livelihood zone . An HEA expresses all cash income and all the food grown and produced by each wealth group as a percentage of the family’s energy specifications based on 2,100 kcal/person/day, irrespective of sex or age. The total amount of energy is estimated to meet the needs of a typical family.

To align the two methods the Cost of the Diet method has selected families of between 4 and 11 individuals who require as close as possible to the total energy requirement of N x 2,100 kcal/person/day specified in an HEA (± 0.3%) in which N is the number of individuals in the family of each wealth group. This allows the Cost of the Diet to use data on income generated by an HEA in order to estimate the affordability of the diet, usually expressed as a percentage of total income. If the energy requirement of a family used to estimate the cost of a diet was greater or smaller than the total energy specification used to estimate a household’s income or expenditure in an HEA, then the affordability of the diet would be either under- or over-estimated. When HEA data are used to estimate the affordability of the cost of a diet, the analysis should apply the standard CotD/HEA family for each wealth group, if different, so that the methods are aligned and data can reliably be compared.

There are other advantages of using these standard families. They can be used to show how the cost of a diet varies with household size for energy set at an average of 2,100 kcal/person/day. This amount of energy per person is also used by United Nations agencies to calculate energy requirements when designing food rations . A Cost of the Diet assessment thus offers a way of calculating a ration based on average energy specification from local foods at the lowest cost. This presumes that local foods are available, which they may not be during an emergency.

**Appendix 5.**  *Factors used to adjust energy requirements*.

Because no standard deviations of average energy intakes were available, a typical coefficient of variation (CV) of 15% has been applied to estimate the standard deviation of the average energy requirement (AVG) and then adjust the amount of energy to a specified percentile (PCT) by applying a mathematical function to calculate the inverse of the standard normal cumulative distribution (INV) as follows:

Energy = EAR + ((AVG * CV) * (INV(PCT/100))

So to calculate the 80^th^ percentile of energy intake for a person with an average energy intake of 2,500 kcal:

Energy = 2500 + (2500 * 0.15) * INV(80/100)

= 2500 + (375 * 0.842)

= 2500 + 316

= 2816 kcal

The default coefficient of variation of 15% cannot be adjusted.

**Appendix 6.** *Factors used to adjust protein requirements*.

The default value in the calculations is the 95^th^ percentile, as recommended by the WHO . The software applies the estimated average requirement (EAR) and standard deviation (SD) of protein per kilogram of body weight specified by the WHO and the inverse of the standard normal cumulative distribution (INV) to calculate the percentile of protein intake per kg of body weight:

Protein (g/kg) = EAR + (SD * (INV(PCT/100)))

The amount of protein is then multiplied by the body weight of adults, which is specified in the values given by the WHO for average energy intake, and ranges from 45-85 kg for women and 50-90 kg for men in 5 kg intervals . The body weight of children is taken from the median values published by the WHO for boys and girls by month of age from zero to 19y of age in the standard growth references . The weight of children of either sex is taken as the midpoint between the median weight of boys and girls of the same age. The body weight of children in a range in age used in the software is calculated from the mid-point in weight of boys and girls at the midpoint in the age range. For example, the weight of a child of either sex aged 12-23 months is taken to be the midpoint between the weight of a boy and a girl aged 18 months published by the WHO .

To calculate the weight of protein required each day by any given individual, the weight of protein required per kilogram is multiplied by body weight:

Protein (g/day) = Protein (g/kg) * body weight (kg)

To calculate the 95^th^ percentile of protein intake for a person weighing 50 kg with a mean requirement of 0.66 g/kg/day and a standard deviation of 0.09 using the following equations:

Protein g/kg = 0.66 + (0.09 * 1.6449)

= 0.66 + 0.148

= 0.808

Protein g/day = 50 * 0.808

= 40.4 g

**Appendix 7.** *Factors used to adjust fat requirements.*

The software applies the following default values for fat requirements.

For children aged 1 to 6 months the minimum amount of fat is based upon the quantity contained in the amount of breast milk required to meet recommended average energy requirements; the maximum is set at 60% of energy specifications for this age group, as recommended .

For children aged between 6 and 23 months it is recommended that the percentage of energy from fat reduces gradually from 40-60% to 25-35% so it has been set at: a minimum of 35% and a maximum of 50% for children aged 6-8 months and 9-11 months; and a minimum of 30% and a maximum of 40% for children aged 12-23 months .

For individuals aged 2 to 18 years it is recommended that a minimum of 25% and a maximum of 35% energy comes from fat .

For adults aged 19 to 60+ years it is recommended that a minimum of 20% of energy and a maximum of 35% of energy comes from fat .

**Appendix 8.** *Factors used to adjust vitamins and minerals requirements*.

For each micronutrient the RNI is divided by a conversion factor (FAC) published by the WHO and FAO to calculate the estimated average requirement (EAR):

EAR = RNI / FAC

Assuming a normal distribution, the standard deviation is thus half the difference between the EAR and the RNI. To calculate the amount equivalent to a percentile, a mathematical function is applied in the software to calculate the inverse of the standard normal cumulative distribution (INV) for the percentile (PCT) expressed as a probability e.g. 50^th^ percentile = 0.5. This function is then used as a multiplier to calculate the amount of nutrient that is equivalent to the percentile of the normal distribution as follows:

Percentile amount = EAR + (INV(PCT/100) * (RNI – ((RNI/FAC))/2)))

For example, to calculate the 80^th^ percentile of a nutrient with an RNI of 400 and a conversion factor of 1.4:

EAR = RNI / FAC = 400 / 1.4 = 286

then: INV(PCT/100) = INV(80/100) = INV(0.8) = 0.84

Percentile amount = 286 + (0.84 * ((400 – (400/1.4)/2))

= 286 + (0.84 * (400 – 286)/2)

= 286 + (0.84 * (114/2)

= 286 + (0.84 * 57)

= 286 + 48

= 334

**Appendix 9.** *Portion sizes*

The default portion size for each food is calculated first as a percentage of average energy intake of a child aged 1-3 years based on 50% of energy from a staple food, 30% from fats, 10% from fruit and vegetables and 10% from protein foods and then divided by three meals a day. For example:

50% of the average energy intake of a child aged 1-3 y =

= 1038.5 * 0.5

= 519 kcal/d

Divided by the average energy density of rice

= 519/2.81

= 184 g/d

Divided into a maximum of three meals

= 184/3

= 61g (rounded to 60g)

The numerator is set at 2 SD above the mean to allow for people with a large energy requirement based on the assumption that they can meet their daily energy needs from food with an average energy density of 1.0 kcal/g. Thus for a food with a portion size of 20 g and for an individual whose energy requirement 2 SD above the mean is 3,750 kcal, the maximum weight of that food per meal is:

Maximum portion size = 20 * (3750/1038.5) = 72 g

**Appendix 10.** *Total weight of food.*

The calculation for the upper limit on food intake differs depending on the age of the individual.

For children under the age of 6 months the maximum weight of breast milk consumed has been calculated by dividing an energy requirement of 2SD above the mean by an average energy density of breast milk of 0.67 kcal/g .

For children aged 6-23 months the maximum weight of food has been calculated as the weight of complementary foods with an assumed and arbitrary low average energy density of 1.0 kcal/g plus the recommended intake of breast milk calculated using an energy density of 0.67 kcal/g .

For children aged 2 - 18 years and all men and women the maximum weight of food has been calculated by dividing an energy requirement that is 2 SD above the mean for each given individual by an assumed but arbitrary low energy density of the diet of 1.0 kcal/g.

**Appendix 11.** *Absorption factors iron.*

The iron absorption factors applied to all foods in the food composition table database are as follows:

| **Food** | **Percentage absorption** | **Factor applied to food** |
| --- | --- | --- |
| Meat, fish, poultry, eggs | 25% | 0.25 |
|  |  |  |
|  |  |  |
| Milk | 11.75% | 0.1175 |
| Plant foods | 5% | 0.05 |
|  |  |  |
| Fats and oils | 25% for animal based fats/oils | 0.25 |
|  | 5% food plant based fats/oils | 0.05 |
| Fortificant or supplemental iron | 2% for elemental iron | 0.02 |
|  | 7% for Fe EDTA, Fe fumarate or Fe sulphate | 0.07 |
|  | 5% for other iron fortificants or if form unknown | 0.05 |

**References**
